# Colocalization of IgG and IgA Heavy Chains with Kappa and Lambda Light Chains in Glomerular Deposits of IgA Nephropathy Patients Using High-Resolution Confocal Microscopy and Correlation with Oxford MEST-C Scores

**DOI:** 10.3390/jcm12237361

**Published:** 2023-11-28

**Authors:** Dana V. Rizk, Lea Novak, Stacy D. Hall, Zina Moldoveanu, Bruce A. Julian, Jan Novak, Mark Haas

**Affiliations:** 1 Department of Medicine, Division of Nephrology, University of Alabama at Birmingham, Birmingham, AL 35294, USA; bjulian@uabmc.edu; 2 Department of Microbiology, University of Alabama at Birmingham, Birmingham, AL 35294, USA; lnovak@uab.edu (L.N.); shall@uab.edu (S.D.H.); zinam@uab.edu (Z.M.); jannovak@uab.edu (J.N.); 3 Department of Pathology & Laboratory Medicine, Cedars-Sinai Medical Center, Los Angeles, CA 90048, USA; mark.haas@cshs.org

**Keywords:** IgA nephropathy, IgAN, immune complexes, light chains, confocal microscopy, MEST-C scores, Oxford classification

## Abstract

Routine immunofluorescence microscopy of glomerular immunodeposits in IgA nephropathy shows IgA, C3, and lambda light chains, and sometimes IgG, IgM, and kappa light chains. However, a previous study using high-resolution confocal microscopy showed IgG in all IgA nephropathy cases, likely representing autoantibodies specific for galactose-deficient IgA1. Here, we used high-resolution confocal microscopy to examine the composition of glomerular immunodeposits and colocalization of kappa and lambda light chains with IgA or IgG heavy chains in kidney-biopsy samples from twenty patients with IgA nephropathy, seventeen without IgG, and nine with no or trace kappa light chains by routine immunofluorescence microscopy. IgG was detected in all biopsies by high-resolution confocal microscopy. Single-optical-plane images showed similar colocalization of IgG heavy chains with kappa and lambda light chains. Colocalization of IgA heavy chains was greater with lambda light chains than with kappa light chains. Colocalization of IgG heavy chain with kappa light chains was higher than with lambda light chains in biopsies with endocapillary hypercellularity and crescents, i.e., biopsies with active lesions. We confirmed the utility of high-resolution confocal microscopy to detect components of glomerular immunodeposits not apparent on routine immunofluorescence microscopy and for colocalization of different components, potentially clarifying the pathogenesis of IgA nephropathy.

## 1. Introduction

There is now substantial evidence supporting a four-hit model of the pathogenesis of IgA nephropathy (IgAN), the most common primary glomerular disease in the world [1,2,3,4]. In this model, galactose-deficient IgA1 (gd-IgA1) in the circulation serves as an autoantigen against which IgG and IgA autoantibodies are formed [3,5,6]. These autoantibodies bind to gd-IgA1 to form circulating immune complexes to which additional proteins may be added; some of the resultant complexes deposit in the mesangium of the glomeruli where they stimulate mesangial-cell proliferation, and induce production of pro-inflammatory mediators [1,3]. This postulated mechanism agrees with the observation that IgA1, in the glomerular immunodeposits, is enriched for gd-IgA1 glycoforms [7,8]. Glomerular immune deposits also contain complement C3 and other complement proteins, consistent with complement activation via the alternative pathway and, in some cases, the lectin pathway [9,10]. The autoantibodies, including IgG autoantibodies, have been detected in the circulation, although kidney biopsies of patients with IgAN demonstrate IgG staining in less than 50% of cases by routine immunofluorescence microscopy [2,5,6,11,12]. However, recent studies using confocal microscopy described detection of glomerular IgG co-deposits in all cases of IgAN studied, and demonstrated that this IgG was specific for gd-IgA1 [13]. Other studies have shown that IgG autoantibodies represent the predominant isotype of circulating antibodies specifically directed against gd-IgA1 in patients with IgAN and that IgG-gdIgA1 immune complexes are pathogenic [5,14].

In addition to the reduced content of galactose in the IgA1 hinge-region *O*-glycans, IgA1 from serum of patients with IgAN has a significantly lower kappa:lambda light-chain ratio than does serum IgA1 from control individuals [15,16,17]. IgA1 with lambda light chains has extensively sialylated *O*-glycans [18]. Notably, this lambda light-chain predominance is often reflected in the relative intensities of kappa and lambda light-chain immunostaining in glomeruli from kidney biopsies showing IgAN, even to the point that some biopsies show strong lambda light-chain staining with only trace or no kappa light-chain staining [2,19]. These findings suggest that the gd-IgA1 in IgAN is lambda-light-chain dominant. Thus, it may be inferred that much of the kappa staining by routine immunofluorescence microscopy in IgAN glomeruli represents IgG autoantibody against gd-IgA1 and that the staining for kappa light chains colocalizes with that for IgG heavy chains.

The aims of this study were to confirm the previously reported presence of IgG in glomeruli from all biopsies, determine if all biopsies also stain for kappa light chain, and examine colocalization of kappa and lambda light chains with that of IgA and IgG heavy chains in the glomeruli [13]. We also examined whether the obtained colocalization data correlated with Oxford MEST-C scores, the validated prognostic light-microscopy features of the kidney biopsies [20].

## 2. Materials and Methods

### 2.1. Patients and Biopsies

Computerized pathology records from the Department of Pathology and Laboratory Medicine, Cedars-Sinai Medical Center (CSMC) in Los Angeles were searched for native-kidney biopsies with a primary diagnosis of IgAN between 1 January 2012 and 31 December 2019. Biopsies selected met the following criteria: 1. The biopsy was performed at CSMC, met Oxford classification criteria for tissue adequacy, and had direct routine immunofluorescence microscopy studies performed on frozen sections of fresh tissue with at least 5 glomeruli in these sections and with remnant frozen tissue being stored at −70 °C since the time of biopsy processing [21]. Biopsies from CSMC (as opposed to outside hospitals) were preferable because clinical parameters at the time of biopsy were more readily available for these patients. 2. The patients had no evidence of a secondary cause of IgAN, including IgA vasculitis (Henoch–Schönlein purpura) or liver disease. A total of 25 biopsies meeting these criteria were identified and each biopsy was assigned a study number. The frozen tissue specimens, with only this study number as a label, were then sent on dry ice to the University of Alabama at Birmingham (UAB) for confocal microscopy analysis. 

At CSMC, two files were created and were kept in a locked drawer until completion of the confocal microscopy studies: one linking each study number with the CSMC surgical pathology number and a second containing demographic and clinical data for each biopsied patient. Data at the time of biopsy included sex, age, serum creatinine concentration, estimated glomerular filtration rate (eGFR) determined using the race-free CKD EPI formula, and proteinuria determined by urine protein/creatinine ratio or 24-h urine protein [22]. Pathologic data including M, E, S, T, and C scores were compiled after review of the biopsy reports using the established Oxford classification criteria [20] (Table 1). All kidney biopsies were read and scored by one pathologist (MH).

### 2.2. Confocal Microscopy and Routine Immunofluorescence Microscopy 

From remnant frozen kidney-biopsy tissues (n = 25) obtained from CSMC, 20 biopsies had glomeruli suitable for confocal microscopy; the remaining 5 biopsies were excluded due to absence of glomeruli in the remnant tissue. These biopsy specimens had been previously examined by routine immunofluorescence microscopy at CSMC, using FITC-conjugated polyclonal rabbit antisera to human IgG, IgA, IgM, C1q, C3, and kappa and lambda light chains, all from Dako/Agilent, Santa Clara, CA, USA. All immunofluorescence results were reported on a scale of 0 to 4+. If the pathologist had reported a score between 2 numbers on the scale, these numbers were averaged, such as an IgA stain of 1-2+ was reported as 1.5. Trace staining was reported as 0.5. All specimens had glomerular IgA deposits (19/20 ≥ 2+ and the other, 1.5). Eighteen biopsies had shown ≥1+ staining for C3 (18/20 ≥ 1+). Only 3 biopsies showed IgG (≥1+). Nineteen biopsies showed >1+ staining for lambda light chains. Three biopsies were negative for kappa light chains while 6 others had shown only trace staining (<1+) for kappa light chains. None of the biopsies had shown glomerular staining for C1q. Only 3 biopsies had more than trace staining for IgM (Table 1). 

For the confocal microscopy studies, 4-μm sections from the 20 remnant frozen kidney-biopsy tissues were cut at UAB. The sections were stained with a nanobody specific for the constant heavy-chain domain 3 (CH3) of human IgG (CaptureSelect IgG-Fc nanobody, Thermo Fisher Scientific, Waltham, MA, USA catalog # 7103262100, dilution 1:500) conjugated to biotin that was subsequently detected with streptavidin-Alexa 555 diluted 1:1500 (*red*) or against IgA (Alexa Fluor 594-conjugated goat anti-human IgA, α chain-specific, Jackson ImmunoResearch, West Grove, PA, USA, catalog # S32355, dilution 1:400, *red*). Biopsies stained for IgA or IgG were then stained with antibody specific for human kappa light chain (Alexa Fluor 488-conjugated anti-human light chain kappa monoclonal antibody, Biolegend, San Diego, CA, USA, clone MHK-49, catalog # 316512, dilution 1:100; *green*), antibody specific for human lambda light chain (Alexa Fluor 647-conjugated anti-human light chain lambda monoclonal antibody, Biolegend, clone MHI-38, catalog # 316614, dilution 1:100; light emission in the far-red spectrum, with color redefined to *blue* for imaging), and nuclear stain (DAPI, Thermo Fisher Scientific, emission 457 nm (*blue*, redefined to *gray* for imaging)). 

Staining protocol: Frozen kidney-biopsy-tissue 4-μm sections on slides were hydrated for 3 min with PBS, fixed in 3.7% paraformaldehyde for 5 min, washed 3× in PBS for 5 min for each wash, blocked with blocking buffer (5% BSA in PBS with 0.05% sodium azide) for 1 h, washed 3× with PBS and blocked with Avidin-Biotin kit from Dako/Agilent (when IgG stain was used) for 15 min each, followed by 3× wash with PBS. Each antibody was incubated with the samples for 2 h at room temperature. Tissues sections were washed 3× with PBS between the application of antibodies and after the last application of antibodies. Slides were cover-slipped and mounted using Vectashield anti-fade mounting media (Vector laboratories, Newark, CA, USA). The slides were wrapped in aluminum foil to prevent fading and stored at +4 °C until used for imaging.

Confocal microscopy was performed using a Nikon A1R/SIM microscope. Images of multiple optical planes were collected (objective 60×) and saved as nd2 files with image denoising. The size bar showed the magnification. For each staining combination (IgA, kappa, lambda or IgG, kappa, lambda), at least 59 regions of interest (ROIs) were selected for colocalization measurement using single optical planes selected from z-stack images. For slides stained with antibodies specific for IgG heavy chain, kappa light chain, and lambda light chain, ROIs were selected in areas exhibiting IgG staining; colocalization data of pairs (IgG-kappa, IgG-lambda) were collected. Similarly, for slides stained with antibodies specific for IgA heavy chain, kappa light chain, and lambda light chain, ROIs were selected in areas exhibiting IgA staining; colocalization data of pairs (IgA-kappa, IgA-lambda) were collected. Colocalization measurements of Pearson’s correlation coefficient were performed using advanced NIS Elements 5.0 Imaging Software, version 5.02 and the data were exported into Excel for statistical analysis. 

### 2.3. Statistical Analysis

Using JMP Pro 16 software version 16.0.0 colocalization ROI mean values for Pearson’s r correlation coefficient were determined for each slide. Then, each slide’s mean value was analyzed by one-way ANOVA for analysis of means to determine significant *p*-values. After unblinding, MEST-C scores for biopsy specimens were incorporated with Pearson’s r for each colocalization group (M0 versus M1, E0 versus E1, S0 versus S1, T0 versus T1/T2, and C0 versus C1/C2) and analyzed by one-way ANOVA for significance. *p*-Values ≤ 0.05 were considered significant. The team performing staining, imaging, colocalization analyses, and statistical evaluation was blinded to the findings of routine immunofluorescence microscopy until all analyses were finalized.

## 3. Results

### 3.1. Kappa and Lambda Light-Chain Colocalization with IgG vs. IgA Heavy Chains

**Figure 1:**
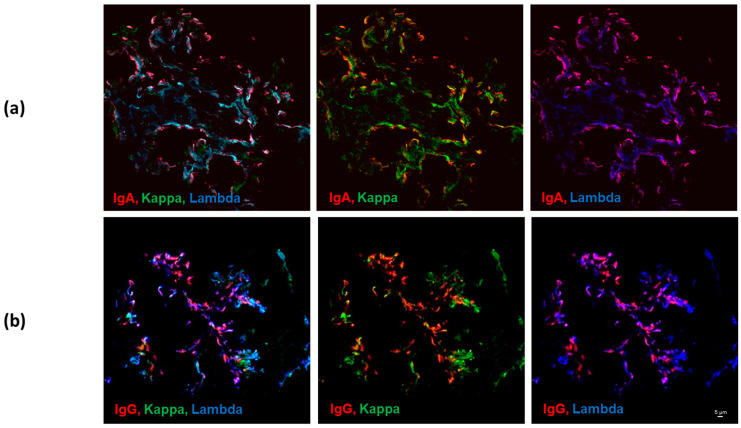
Examples of confocal-microscopy images of glomeruli in remnant kidney-biopsy tissue from patients with IgAN stained for IgG or IgA and kappa and lambda light chains. Images in a single optical plane show colocalization of components of immune complexes: IgA, red; kappa light chain, green; lambda light chain, blue (**a**); and IgG, red; kappa light chain, green; lambda light chain, blue (**b**). Combinations of colors shown are noted in each panel. Objective = 60×, size bar = 5 μm (shown in the panel (**b**), same for all panels).

**Figure 2:**
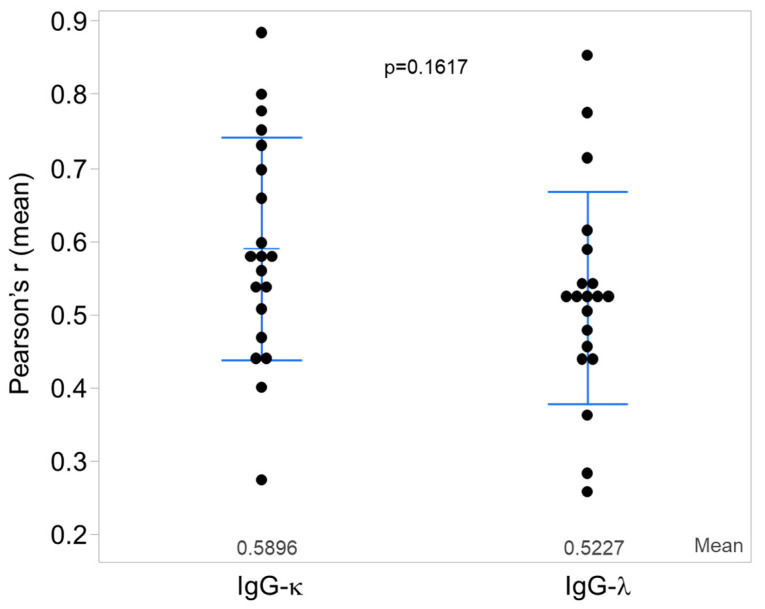
Statistical analysis of colocalization of IgG with kappa (κ) and lambda (λ) light chains. Mean values of Pearson’s r colocalization for IgG-κ and IgG-λ for each patient (n = 20) are shown. Bars show mean values ± SD. Colocalization of IgG-κ and IgG-λ did not show a statistically significant difference (*p* = 0.1617).

Although only three of the twenty biopsies examined showed glomerular deposits of IgG heavy chain by routine immunofluorescence microscopy, all biopsies showed the presence of IgG heavy chain by confocal microscopy. Likewise, although three biopsies showed no staining for kappa light chain and six others only trace kappa light-chain staining by routine immunofluorescence microscopy, by confocal microscopy all twenty biopsies showed the presence of kappa light chains. Both kappa and lambda light chains colocalized with glomerular IgG and IgA heavy-chain deposits by confocal microscopy. Figure 1 shows examples of single-optical-plane confocal-microscopy images of glomeruli stained for components of immune complexes: IgA heavy chain, kappa, and lambda light chains (Figure 1, panel a), and IgG heavy chain, kappa, and lambda light chains (Figure 1, panel b). Colocalization of IgG heavy chain was not significantly different with kappa versus lambda light chains (Figure 2). 

**Figure 3:**
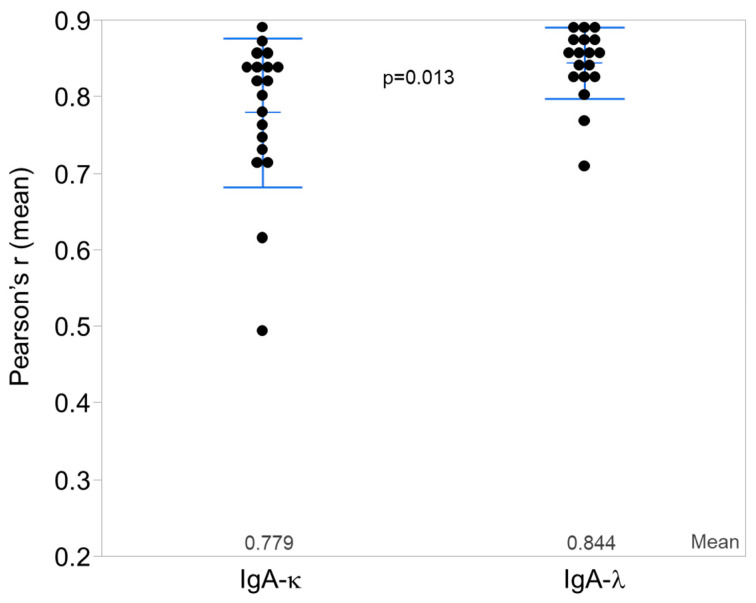
Statistical analysis of colocalization of IgA with kappa (κ) and lambda (λ) light chains. Mean values of Pearson’s r colocalization for IgA-κ and IgA-λ for each patient (n = 20) are shown. Bars show mean values ± SD. IgA-λ colocalization was significantly higher compared with IgA-κ (*p* = 0.013).

Although both kappa and lambda light chains colocalized with IgA heavy chain in the glomerular deposits, colocalization was significantly greater for IgA heavy chain with lambda light chain than for IgA heavy chain with kappa light chain (Figure 3). Supplemental Table S1 shows individual-level data for colocalization of IgG or IgA heavy chains with kappa or lambda light chains.

### 3.2. Correlation of Light Chain—Heavy Chain Colocalization with Oxford MEST-C Scores

Table 2 summarizes the MEST-C scores stratified by results from routine immunofluorescence microscopy for kappa and lambda light chains in the glomeruli. In this limited sample set, these scores in the full group of 20 biopsies did not differ from those for the 11 biopsies showing more than trace (≥1+) glomerular staining for kappa light chains or for the 19 biopsies showing more than trace (≥1+) glomerular staining for lambda light chains. Table 3 shows the colocalization of glomerular IgG heavy chain with kappa light chains or lambda light chains as a function of the MEST-C scores for each biopsy. While IgG heavy chain colocalization with lambda light chain did not differ between biopsies when grouped (M0 versus M1, E0 versus E1, S0 versus S1, T0 versus T1/T2, and C0 versus C1/C2), the colocalization of IgG heavy chain with kappa light chain was significantly greater in biopsies with E1 versus E0 (*p* = 0.050) and C1/C2 versus C0 (*p* = 0.033). For colocalization of glomerular IgA heavy chain and light chain staining, the only significant association with MEST-C scores was a greater colocalization of IgA heavy chain with lambda light chain in biopsies with C0 compared to those with C1/C2 (*p* = 0.048; Table 4).

## 4. Discussion

In this study, native-kidney biopsies of patients with IgAN were examined using confocal microscopy and very sensitive reagents for staining. We compared the findings with the results of routine immunofluorescence microscopy. Specifically, we evaluated colocalization of IgG or IgA heavy chains with kappa and lambda light chains. We correlated these results with the prognostic Oxford classification histological scores. Analysis of IgA1 in serum and glomeruli of patients with IgAN has shown a predominance of lambda light chain compared with kappa light chain [15,19,23,24]. Our finding that glomerular IgA heavy chain showed greater colocalization with lambda than kappa light chains is consistent with these results. A recent mass spectrometry-based profiling revealed distinct clonal repertoires of the J-chain-coupled polymeric IgA1 and monomeric IgA1 [25]. This discovery poses a question whether the lambda light chain predominance in the IgA1 of immunodeposits in IgAN is associated with polymeric gd-IgA1. Furthermore, what genetic factors and immunological and biochemical pathways are involved in the production of this autoantigen [26,27,28,29,30,31,32]?

The findings by confocal microscopy showed the presence of glomerular IgG heavy chain in all twenty cases of IgAN, even though only three biopsies had IgG heavy chain detected by routine immunofluorescence microscopy. This observation confirmed findings from a previous study that detected IgG heavy chain in the glomerular immune deposits in all cases of IgAN with use of a nanobody specific for IgG heavy chain and confocal microscopy [13]. Consistent with this finding, staining for kappa light chains was present in all cases by confocal microscopy, even in those with no or only trace kappa light chain staining by routine immunofluorescence microscopy. Both kappa and lambda light-chain staining colocalized with that for IgG heavy chain, consistent with the presumed polyclonal nature of these autoantibodies [12]. Moreover, we found that IgG-kappa colocalization within these deposits was significantly greater than IgG-lambda colocalization in biopsies with active histologic lesions, namely endocapillary hypercellularity (Oxford score E1) and crescents (Oxford score C1/C2). In contrast, IgA-lambda colocalization was greater in biopsies with C0 than in those with C1/C2. These associations between light- and heavy-chain colocalization and MEST-C scores might be interpreted as suggesting greater concentration of autoantibody (IgG-kappa) within glomerular immune complex deposits in more active lesions of IgAN. However, these data are difficult to interpret and examination of more biopsies might clarify these findings in the future.

Other colocalization studies based on specialized staining and confocal microscopy have shown that IgA and complement C3 are not always evenly distributed in the immunodeposits, and that IgA or C3 can coat surfaces of these immunodeposits [33]. This study found that the patterns of spatial organization of IgA and C3 correlated with glomerular damage. Specifically, kidney-biopsy tissues with mild to moderate diffuse mesangial proliferation had immunodeposits coated by an outer layer of IgA. Conversely, biopsies with diffuse proliferative endocapillary and/or extracapillary patterns contained deposits without an outer coat of IgA. Another study assessed the presence of abnormally glycosylated IgA1 in the immunodeposits and its spatial relationship with complement C3 [34]. This study correlated these features with glomerular-lesion severity and essentially confirmed the findings of the earlier study [33]. The authors concluded that spatial organization of IgA and C3 impacts glomerular injury [34]. In the present study, we have for the first time used confocal microscopy to analyze colocalization of kappa and lambda light chains with IgA or IgG heavy chains in the glomeruli. IgG, the driver of immune-complex formation, was detected in the samples from all patients, confirming our prior observations [13].

Studies examining the association between glomerular light-chain and IgG heavy chain staining by routine immunofluorescence microscopy or immunohistochemistry and histologic and clinical findings in IgAN have yielded divergent results. In a recent study from China, IgAN patients with lambda light-chain-restricted glomerular deposits had more proteinuria, mesangial and endocapillary hypercellularity, crescents, and T1/T2 lesions (as opposed to T0) than IgAN patients whose biopsies did not show light-chain-restricted deposits [35]. The former patients also had a higher likelihood of reaching a composite endpoint of 30% decrease in eGFR, kidney failure, or death. However, a study from the NIH CureGN consortium found no difference in proteinuria or outcomes between IgAN patients whose findings on routine immunofluorescence microscopy showed lambda-dominant glomerular deposits versus those with a difference of kappa and lambda light-chain staining intensities of <1+ [36]. The lambda-dominant biopsies showed more intense staining for IgG heavy chain and a higher fraction of biopsies with E1 lesions but there was no difference between the two groups with respect to the Oxford classification M, S, T, and C scores [36]. In the original Oxford IgAN study cohort, the presence of glomerular IgG heavy-chain deposits (>trace) was found in 20% of biopsies for which this information was available. This finding was associated with higher frequency of mesangial hypercellularity and endocapillary hypercellularity, with a trend toward worse survival from a combined event of kidney failure or a ≥50% reduction in initial eGFR compared to cases with no or trace IgG heavy chain; these results are consistent with some previous reports [11,37]. The observations in the Oxford IgAN cohort appear consistent with an association of detectable IgG heavy chain autoantibody within glomerular deposits and more severe disease. However, the findings are based on a rather insensitive method for detecting IgG heavy chain. The current study, using nanobody specific for IgG heavy chain, confirmed an earlier report of glomerular IgG detected by confocal microscopy in all cases of IgAN.

## 5. Conclusions

We detected IgG heavy chain in the glomerular immune-complex deposits in all IgAN biopsies, even though most biopsies had not shown glomerular staining for IgG heavy chain by routine immunofluorescence microscopy [13]. This finding strengthens the postulated role of IgG autoantibody in the kidney injury in all patients with IgAN. IgG heavy chain colocalized with staining for both kappa and lambda light chains, as would be expected for a polyclonal autoantibody. We postulate that much of the kappa staining by routine immunofluorescence microscopy in IgAN glomeruli represents IgG autoantibody against gd-IgA1. By contrast, colocalization of glomerular IgA heavy chain was significantly greater with lambda than kappa light chain, consistent with the lambda light chain predominance of IgA1 in patients with IgAN [15,17,37]. Greater colocalization of IgG heavy chain with kappa versus lambda light chain in glomerular deposits was associated with the presence of active glomerular lesions, namely Oxford MEST-C scores E1 and C1/C2. The significance of this last observation will need to be evaluated in future studies.

## Figures and Tables

**Table 1:** Demographic, clinical, and pathologic characteristics at the time of diagnostic kidney biopsy.

Subject	Age (Years)	Gender	SCr (mg/dL)	eGFR (mL/min/1.73 m^2^)	UPCR or 24-h Urinary Protein	Oxford Classification					Routine Immunofluorescence Staining					
						M	E	S	T	C	IgA	IgG	IgM	C3	κ	λ
1	37	M	1.2	80	1.1 g/g	0	1	0	0	1	4	0	0	4	1.5	2
2	59	M	NA	NA	NA	0	0	0	0	0	3.5	0	0.5	2.5	1.5	2.5
3	24	M	1.0	108	1.2 g/g	1	1	1	0	1	3	0	0.5	1.5	0	2.5
4	54	F	11.8	3	NA	1	1	0	1	1	2.5	0	0	2	0.5	1.5
5	32	M	0.9	116	0.8 g/g	1	0	1	0	0	4	0	0	3	2.5	3
6	32	F	1.7	41	4.8 g/d	0	0	1	2	0	2.5	0	0.5	0.5	0.5	1.5
7	33	M	1.3	74	0.7 g/g	0	1	1	0	1	4	0	0	1.5	2	2.5
8	25	M	1.2	86	0.8 g/g	1	1	0	0	1	4	0	0	4	0.5	3.5
9	42	F	2.2	28	NA	1	0	0	0	0	2	0	0	1.5	0	1.5
10	48	M	1.5	57	2.0 g/d	1	1	1	1	1	4	3	2	3	3	3
11	29	F	1.0	78	NA	0	0	1	0	1	3	1	0	2.5	0.5	2.5
12	40	F	0.6	116	4.5 g/g	1	0	1	0	1	4	0	0	4	2.5	2.5
13	29	M	1.1	93	1.0 g/d	1	0	1	0	0	3	1	0	2	1	2.5
14	38	F	1.6	42	1.3 g/g	0	0	1	1	0	4	0	1	3	3	4
15	67	M	1.2	66	NA	0	0	0	0	0	1.5	0	0	1	0.5	0.5
16	40	M	1.2	78	1.0 g/g	1	1	1	0	1	4	0	0	3.5	2	3
17	54	M	1.7	47	1.3 g/g	1	1	1	0	1	2	0	0	3	0.5	1.5
18	61	F	1.4	43	0.4 g/d	1	0	0	1	0	4	0	0	2.5	2	3
19	48	F	2.5	23	1.8 g/g	1	0	1	1	0	2.5	0	0	3	0	1.5
20	21	M	1.2	88	0.3 g/g	1	1	1	0	0	4	0	1	4	2	2

SCr, serum creatinine; eGFR, estimated glomerular filtration rate; UPCR, urine protein-to-creatinine ratio (g/g) in grams of protein per gram of creatinine; 24-h urine protein (g/d), 24-h urine protein in grams per day; M, mesangial hypercellularity; E, endocapillary hypercellularity; S, segmental sclerosis; T, tubulointerstitial fibrosis; C, crescents; IgA, immunoglobulin A; IgG, immunoglobulin G; IgM, immunoglobulin M; κ, kappa light chain; λ, lambda light chain.

**Table 2:** MEST-C scores in the 20 biopsies examined by confocal microscopy.

Biopsies	M0/M1	E0/E1	S0/S1	T0/T1/T2	C0/C1/C2
All (20)	7/13	11/9	7/13	14/5/1	10/10/0
Kappa ≥ 1+ *	4/7	6/5	3/8	8/3/0	7/4/0
Lambda ≥ 1+ *	6/13	10/9	6/13	13/5/1	9/10/0

* = By routine IF microscopy.

**Table 3:** Correlation of IgG-κ and IgG-λ colocalization with MEST-C scores.

	Pearson’s r IgG-κ							Pearson’s r IgG-λ						
	n	x	SD	n	x	SD	*p*-Value	N	X	SD	n	x	SD	*p*-Value
Oxford Score	Score 0			Score 1 (or T1/T2) (C1/C2)				Score 0			Score 1 (or T1/T2) (C1/C2)			
M	7	0.559	0.132	13	0.606	0.164	0.525	7	0.470	0.137	13	0.551	0.146	0.242
E	11	0.539	0.142	9	0.652	0.147	**0.050**	11	0.505	0.107	9	0.544	0.186	0.562
S	7	0.604	0.130	13	0.582	0.167	0.762	7	0.518	0.111	13	0.525	0.164	0.916
T	14	0.590	0.159	5	0.612	0.150	0.964	14	0.504	0.145	5	0.593	0.146	0.594
C	10	0.528	0.127	10	0.651	0.154	**0.033**	10	0.529	0.082	10	0.517	0.193	0.854

M, mesangial hypercellularity; E, endocapillary hypercellularity; S, segmental sclerosis; T, tubulointerstitial fibrosis; C, crescents; IgG, immunoglobulin G; κ, kappa light chain; λ, lambda light chain. Significant *p*-Values in bold and underlined. n: number of subjects; x: mean; SD: standard deviation (±1 SD).

**Table 4:** Correlation of IgA-κ and IgA-λ colocalization with MEST-C scores.

	Pearson’s r IgA-κ							Pearson’s r IgA-λ						
	n	x	SD	n	x	SD	*p*-Value	n	x	SD	n	x	SD	*p*-Value
Oxford Score	Score 0			Score 1 (or T1/T2) (C1/C2)				Score 0			Score 1 (or T1/T2) (C1/C2)			
M	7	0.756	0.131	12	0.799	0.077	0.385	7	0.843	0.043	12	0.849	0.054	0.829
E	10	0.787	0.115	9	0.779	0.085	0.881	10	0.850	0.064	9	0.843	0.027	0.751
S	7	0.782	0.072	12	0.784	0.115	0.978	7	0.851	0.030	12	0.844	0.059	0.784
T	13	0.800	0.076	5	0.797	0.071	0.951	13	0.847	0.051	5	0.845	0.054	0.939
C	9	0.783	0.122	10	0.784	0.081	0.983	9	0.867	0.044	10	0.829	0.049	**0.048**

M, mesangial hypercellularity; E, endocapillary hypercellularity; S, segmental sclerosis; T, tubulointerstitial fibrosis; C, crescents; IgA, immunoglobulin A; κ, kappa light chain; λ lambda light chain. Significant *p*-Values in bold and underlined. n: number of subjects; x: mean; SD: standard deviation (±1 SD).

## Data Availability

Not relevant to the current work.

## Supplementary Materials

The following supporting information can be downloaded at: https://www.mdpi.com/article/10.3390/jcm12237361/s1, Table S1: Pearson’s r values for colocalization of IgG or IgA with kappa or lambda light chains for individual samples.
