# Supplementary material for: Colocalization of IgG and IgA Heavy Chains with Kappa and Lambda Light Chains in Glomerular Deposits of IgA Nephropathy Patients Using High-Resolution Confocal Microscopy and Correlation with Oxford MEST-C Scores
Source: J Clin Med. 2023 Nov 28;12(23):7361. doi: 10.3390/jcm12237361 (PMC10707091; doi:10.3390/jcm12237361)
Supplement: Supplementary file 1 [file jcm-12-07361-s001.zip › jcm-2697624-supplementary.pdf]

Table S1. Pearson's r values for colocalization of IgG or IgA with kappa or lambda light chains for individual samples.

**Sample ID #**	**Pearson's r IgG**		**Pearson's r IgA**	
	kappa	lambda	kappa	lambda
1	0.400 (0.316)	0.362 (0.308)	0.711 (0.159)	0.802 (0.182)
2	0.580 (0.257)	0.448 (0.286)	0.801 (0.123)	0.848 (0.117)
3	0.884 (0.086)	0.614 (0.230)	0.615 (0.170)	0.876 (0.080)
4	0.730 (0.199)	0.504 (0.265)	0.715 (0.132)	0.844 (0.080)
5	0.535 (0.234)	0.522 (0.253)	0.779 (0.148)	0.858 (0.102)
6	0.468 (0.325)	0.435 (0.289)	0.494 (0.181)	0.848 (0.099)
7	0.697 (0.154)	0.519 (0.189)	0.836 (0.109)	0.872 (0.098)
8	0.658 (0.164)	0.478 (0.268)	0.758 (0.147)	0.828 (0.120)
9	0.777 (0.117)	0.588 (0.223)	0.735 (0.124)	0.860 (0.095)
10	0.800 (0.177)	0.853 (0.158)	0.859 (0.071)	0.823 (0.151)
11	0.751 (0.151)	0.283 (0.280)	0.831 (0.110)	0.874 (0.073)
12	0.438 (0.177)	0.519 (0.230)	0.843 (0.077)	0.709 (0.141)
13	0.274 (0.310)	0.442 (0.314)	NA	NA
14	0.441 (0.321)	0.530 (0.268)	0.730 (0.130)	0.768 (0.129)
15	0.577 (0.218)	0.713 (0.177)	0.890 (0.069)	0.890 (0.089)
16	0.597 (0.179)	0.775 (0.137)	0.824 (0.107)	0.824 (0.123)
17	0.559 (0.236)	0.258 (0.317)	0.841 (0.072)	0.835 (0.088)
18	0.507 (0.202)	0.532 (0.228)	0.866 (0.077)	0.883 (0.073)
19	0.580 (0.176)	0.544 (0.204)	0.815 (0.141)	0.908 (0.084)
20	0.539 (0.221)	0.535 (0.221)	0.853 (0.097)	0.880 (0.107)

Pearson's r values are shown as mean (+/- SD) values.

NA: not analyzed, sample without glomerulus.
